# Thermal Vulnerability of Reef Fish and Implications for Subsistence Fisheries Throughout the Caribbean

**DOI:** 10.1111/gcb.71118

**Published:** 2026-10-06

**Authors:** Mary R. Cortese, Steven W. J. Canty, Sara M. Melo‐Merino, A. Justin Nowakowski

**Affiliations:** ^1^ Smithsonian Environmental Research Center Edgewater Maryland USA; ^2^ Tennenbaum Marine Observatories Network and MarineGEO Smithsonian Institution Edgewater Maryland USA; ^3^ Life on a Sustainable Planet Postdoctoral Fellow, MarineGEO Program Smithsonian Environmental Research Center Edgewater Maryland USA

**Keywords:** coral reef fish, food security, global change, population trends, range shifts, species distribution models, thermal physiology

## Abstract

Dual biodiversity and climate crises have exacerbated declines in global fisheries, resulting in food insecurity for communities that depend on subsistence fishing. As fish populations change and shift their geographic distributions under a changing environment, long‐term planning of fisheries management becomes critical for ensuring food security. Here, we first present physiologically informed species distribution models (pSDM) of seven finfish species in the Caribbean that are important for subsistence in some coastal areas. These models identified areas at risk of climate‐driven declines and other areas of increased suitability and potential refugia, highlighting how shifts in climate suitability for key finfish species may affect long‐term food security in coastal communities. The pSDMs projected mean reductions in climate suitability that ranged from 20% to 66% across all species and scenarios, with more severe decreases under high warming scenarios and in models informed by mean thermal limits across life stages. We then examined whether climate vulnerability estimated from our physiological SDMs explains spatial variation in population biomass trends in the Mesoamerican Reef region using long‐term monitoring data. This analysis suggests that changing climate suitability is already associated with spatial shifts in biomass of several species, reducing resource availability for subsistence fisheries in some areas. These models highlight the potential for substantial future shifts in distributions of species important for subsistence in the coming decades. Information on changing climate suitability can support regional planning efforts to help bolster food security and climate resilience within coastal communities.

## Graphical abstract

We present species distribution models of seven finfish species that are important for coastal subsistence. Models highlight potential for substantial future shifts in distributions in the coming decades. Information on changing climate suitability can support regional planning including marine protected area expansion to help bolster food security and climate resilience.

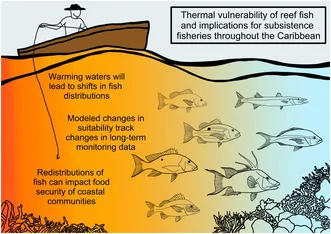

## RESUMEN

Las crisis simultáneas de biodiversidad y climática han agravado el declive de las pesquerías a nivel global, lo que resulta en inseguridad alimentaria para las comunidades que dependen de la pesca de subsistencia. A medida que las poblaciones de peces cambian y desplazan sus distribuciones geográficas en respuesta a un ambiente cambiante, la planificación a largo plazo del manejo pesquero se vuelve fundamental para garantizar la seguridad alimentaria. Aquí, primero presentamos modelos de distribución de especies informados fisiológicamente (pSDM) para siete especies de peces óseos en el Caribe que son importantes para la subsistencia en algunas zonas costeras. Estos modelos identificaron áreas en riesgo de disminuciones provocados por el clima, así como zonas con mayor idoneidad climatica y potenciales refugios, destacando cómo los cambios en la idoneidad climática de especies clave de peces óseos pueden afectar la seguridad alimentaria de las comunidades costeras a largo plazo. Los pSDMs proyectaron reducciones promedio en la idoneidad climática de entre 12% y 66% en todas las especies y escenarios, con disminuciones más severas bajo escenarios de mayor calentamiento y en los modelos informados por los límites térmicos promedio considerando múltiples etapas del ciclo de vida. Posteriormente, examinamos si la vulnerabilidad climática estimada mediante nuestros pSDMs explicaba la variación espacial en las tendencias de biomasa poblacional en la región del Arrecife Mesoamericano, usando datos de monitoreo a largo plazo. Este análisis sugiere que los cambios en la idoneidad climática ya están asociados con desplazamientos espaciales en la biomasa de varias especies, reduciendo la disponibilidad de recursos para la pesca de subsistencia en algunas zonas. Estos modelos destacan el potencial de cambios significativos en la distribución de especies importantes para la subsistencia en las próximas décadas. La información sobre los cambios en la idoneidad climática puede contribuir a los esfuerzos de planificación regional para fortalecer la seguridad alimentaria y la resiliencia climática de las comunidades costeras.

## 1 Introduction

Many coastal fisheries have steadily declined for decades (Pauly and Zeller 2016) as a result of overfishing and other global change drivers. While industrial catch is often exported, small‐scale and subsistence fisheries are critical to food security within coastal communities (Pauly 2018), especially in the developing world. Within the tropics, small‐scale and subsistence fisheries are typically centered around nearshore reefs and coastal wetlands (Ermgassen et al. 2021; Martin et al. 2025). Tracking landed catch trends in small‐scale fisheries is often difficult due to a lack of reporting, which can lead to conflicting reports on the status of fisheries, highlighting the uncertainty around global trends within this important segment of global food production (Zeller et al. 2015). Despite this uncertainty, per capita yield is decreasing as human populations within coastal communities increase (Canty and Deichmann 2022). Pressure from growing coastal populations calls into doubt whether resource availability within small‐scale and subsistence fisheries can meet food security needs for coastal communities, especially as fisheries are threatened by overfishing, coastal development, and climate change in many regions (Cánovas‐Molina and García‐Frapolli 2022; Canty and Deichmann 2022; Damasio et al. 2020; Pauly and Zeller 2016).

Continued access to fish resources is another key aspect of food security, and is particularly important for subsistence fishers, who have limited mobility (Damasio et al. 2020; Daw 2008). Subsistence fishers depend heavily on nearshore, shallow water fishing grounds, including coral reef habitats that are associated with increasing temperatures (Zhu et al. 2014). Most coral reef fish targeted by subsistence fisheries are ectotherms, making them particularly vulnerable to ocean warming (Poloczanska et al. 2016). Species responses to warming depend, in part, on their thermal physiology and adaptations to past and current thermal regimes (Donelson et al. 2019). The sublethal and lethal impacts of warming on individuals and populations of fish can ultimately scale up to shifts in geographic ranges of species over time, reducing the availability and accessibility of preferred species for fishers. As oceans warm, many species are undergoing range shifts (Lenoir et al. 2020), tracking their thermal niche (Sorte et al. 2010; Sunday et al. 2012), which may push some preferred species beyond the conventional fishing grounds of subsistence fishers. These shifts in distributions can also cause restructuring of ecological communities, through changing abundances (Poloczanska et al. 2016) and eventually the addition or removal of key species (Alexander et al. 2015). Species distribution models (SDMs) are commonly used to understand and predict potential range shifts under global change conditions, but very few distribution models explicitly account for physiological thermal limits, leading to projections of species ranges outside what is ecologically plausible (Brewer et al. 2016). Explicit incorporation of thermal tolerances within SDMs allows for more ecologically meaningful and realistic outputs to use within management planning (Feng et al. 2020).

Range shifts can have large impacts on local socio‐economic systems and management projects centered around marine ecosystems (Madin et al. 2012) with species redistribution intersecting with 11 of the UN Sustainable Development Goals including SDG 2 zero hunger (Pinsky et al. 2021). For fisheries species, range shifts can alter catch compositions and lead to asynchrony between fish distributions and relevant regulations, leading to increased conflicts within and between fisheries sectors (Palacios‐Abrantes et al. 2022; Pinsky et al. 2021). Marine Protected Areas (MPAs) are one proposed solution to address food security risk from marine range shifts and biodiversity loss (Nowakowski et al. 2023; Sala et al. 2021). However, many MPAs still allow for some level of fishing (Skinner et al. 2026). Additionally, in areas exposed to greater rates of warming and temperature extremes, climate change can counteract the mitigating benefits of MPAs (Canty et al. 2024). Therefore, implementation of MPAs as a mitigation strategy will be more effective when they are designed and established as climate‐resilient networks (Queirós et al. 2021), further highlighting the need to integrate ecologically realistic assessments of climate vulnerability into marine spatial planning.

Within the Caribbean, small‐scale and subsistence fisheries are culturally and economically important to the region, supporting food security and livelihoods for millions of people (Canty and Deichmann 2022; Price 1966; Salas et al. 2007). Fisheries in the Caribbean face similar pressures as other regions around the world including rapid environmental changes and stock declines, but artisanal fishers are especially vulnerable to fisheries collapse due to livelihood and income dependency upon these resources (FAO 2010). Landings for these fisheries are rarely reported leading to gaps within data and management plans. To fill these gaps, catch reconstruction methods have been used to provide estimates including species used for subsistence (Pauly and Zeller 2016). Using these reconstructions in combination with a recent participatory mapping study in Belize (Wade et al. 2026), we focus on seven species that fishers identified as preferred for consumption within the subsistence fishery, providing a unique opportunity to better understand key species within these understudied fisheries.

Here, we first used a physiologically informed modeling framework to understand how climate suitability for subsistence fisheries is changing across spatial and temporal scales, historical and future, and across key subsistence species with different thermal tolerances throughout the Caribbean. These species include barracuda (Sphyraenidae), white grunt (Haemulindae), hogfish (Labridae), and four species of snapper (Lutjanidae). We then assessed if predicted shifts in regional climate suitability help explain spatial variation in trends in fish abundances throughout the Mesoamerican Reef region (MAR) based on long‐term monitoring data. This work highlights the importance of accounting for thermal physiology within distribution models, especially when using model outputs for management applications.

## 2 Methods

### 2.1 Projecting Changes in Temperature Suitability With Physiologically Informed SDMs

To assess changes in climate suitability for subsistence fisheries we focused on the seven species of finfish listed as “important”—that is, species were consistently listed as a preferred species for fishers within participatory mapping and household surveys (Wade et al. 2026). These species included: *Haemulon plumierii* (white grunt), *Lachnolaimus maximus* (Hogfish), *Lutjanus analis* (mutton snapper), *L. griseus* (gray snapper), *L. synagris* (lane snapper), *Ocyurus chrysurus* (yellowtail snapper), and *Sphyraena barracuda* (great barracuda). We obtained occurrence data for each species from the Global Biodiversity Information Facility (GBIF) (GBIF 2024a, 2024b, 2024c, 2024d, 2024e, 2024f, 2024g) and Ocean Biodiversity Information System (OBIS) (OBIS 2024a, 2024b, 2024c, 2024d, 2024e, 2024f, 2024g), cleaned to remove incomplete identity and georeferenced records, and spatially thinned to include one occurrence point per 5 km^2^ using *spThin* (Aiello‐Lammens et al. 2015) to match spatial resolution of environmental data and reduce potential spatial autocorrelation (Guisan et al. 2017). Downloaded occurrence records were limited to 2000–2024 to more closely align with the temporal resolution of our “current day” climate data. We then combined occurrence records of all seven species and created a 100‐km buffer around the maximum extent of all combined points to develop a maximum area of occurrence in which to crop environmental data and build models (Figure S1).

Here, we modeled species‐specific changes in sea‐surface temperature (SST) suitability—both from recent past to current conditions and projected to end‐of‐century. To characterize “current” temperature conditions, we downloaded monthly maximum SST data at 5‐km resolution between 2005 and 2024 from Coral Reef Watch (NOAA 2019). We then calculated the mean monthly maximum temperature across the past 20 years, which was used as our “current day” temperature scenario. We fit SDMs using these data to estimate suitability as a function of regular exposure to high temperatures for a given area—here, using mean monthly maximum SST. We also used the Coral Reef Watch data (NOAA 2019) from 1985 to 2004 to calculate a 20‐year mean monthly maximum SST to represent the prevailing temperature conditions of the “recent past.” These data were processed in the same way as the “current day” data and were used to back‐project probability of occurrence in the same way our future data was used to project into the future (described below). Future climate scenario data were downloaded from Bio‐Oracle (Assis et al. 2024; Tyberghein et al. 2012) for end of century, 2090–2100, at a spatial resolution of 0.05 degrees (~5 km). We focused on three future change scenarios, including Shared Socioeconomic Pathway (SSP) 2‐4.5, a best‐case scenario with large carbon mitigation efforts undertaken; SSP4‐6.0, a moderate scenario with some mitigation efforts; and SSP5‐8.5, a worst‐case business‐as‐usual scenario. For each scenario, we downloaded the mean monthly mean SST across the 10 year projected time period (2090–2100). We compared distributions of current day mean monthly mean temperature layers from the two data sources to ensure consistency between data products, which were nearly identical (Figure S2). To account for differences between summary metrics (mean monthly mean vs. mean monthly max SST), future layers were adjusted using an additive bias correction (Figure S3). This correction added the difference between current and future mean monthly mean bio‐oracle data to the current day mean monthly max from Coral Reef Watch data—creating future scenario layers for the same temperature metric as the current day Coral Reef Watch data. This bias correction assumes stationarity between current and future temperature distributions. All temperature data were clipped to within the 100‐km buffered area around all presence records for the focal species. While SST can vary from water temperature on the reef, it is the most commonly used temperature variable when running SDMs of fish (Pickens et al. 2021).

To build our pSDMs, we generated points to characterize the background temperature conditions within and just beyond (100 km) the extent of occurrence points. Because we were interested in modeling suitability across the entire thermal niche, we sampled the background temperatures within the buffered occurrence area in a stratified random pattern across environmental space. To ensure the background temperature space was well sampled, we generated an even number of points within one‐degree temperature bins, and within these bins, the points were randomly dispersed across geographic space (Barbet‐Massin et al. 2012; Feng et al. 2020). When compared to other methods for pseudo‐absence generation, including fully random, stratified‐random point generation across environmental space had the highest performance metrics in a previous assessment (Steen et al. 2024). While true pseudo‐absences are built to select areas where the species of interest are more likely to be absent, the terms “pseudo‐absence” and “background sampling” are often used interchangeably (Liang et al. 2018; Sillero et al. 2021; Sillero and Barbosa 2021). We will use the terms as such from here on out. Additionally, our model outputs follow traditional interpretation of background sampling with outputs interpreted as relative probability of occurrence (Merow et al. 2013). We generated an even number of pseudo‐absences as the occurrence points for each species (Barbet‐Massin et al. 2012; Feng et al. 2020). Pseudo‐absences were created using the *adehabitatHR* package (Calenge 2024) across the entire area included in the 100‐km buffer around all occurrence points, assuming that our finfish species were not dispersal limited in the way other benthic or invertebrate species are (Barbet‐Massin et al. 2012; Wisz and Guisan 2009). Our goal was to ensure that background points captured temperature conditions outside those of occurrence points to adequately model upper and lower limits of the thermal niche. Additionally, it has been shown that physiology informed species distribution models are less sensitive to choices of calibration area than non‐physiology informed models (Zhang et al. 2024).

To model the climate suitability of the selected finfish species, we used a Bayesian implementation of a plateau SDM (Brewer et al. 2016; Feng et al. 2020). Whereas a traditional SDM can boundlessly project across environmental space, the plateau allows us to explicitly account for the physiological upper thermal limit of each species within the model—thereby generating more ecologically realistic projections by restricting estimated climate suitability at higher temperatures by the physiological limits of the species. The implementation of the plateau is similar to logistic regression, except that the linear predictor function is constructed with three connecting segments with different slopes, rather than a single linear regression segment (Feng et al. 2020). For comparison and to evaluate the influence of the physiological prior (see below), we fit both physiologically informed and non‐physiological versions of the plateau model. For the non‐physiological plateau model, we specified noninformative priors drawn from a normal distribution for all model parameter estimates. From this non‐physiological model, we extracted the minimum training presence (MTP), a commonly used threshold representing the lowest relative probability value associated with known presence locations (Feng et al. 2020; Pearson and Dawson 2003). For the physiologically informed model, the prior for the *x*‐intercept for the right part of the curve describing probability of occurrence as a function of SST was informed by the species' CT_max_, where CT_max_ in addition to the extracted MTP value acted as an imposed constraint on the parameter space of the model, directing the right segment of the plateau through the intersection of the MTP and the designated thermal max (Figure S4). This prior therefore forced parameter estimates to reflect this marginal condition—restricting predictions of occurrences at temperatures that exceeded this physiological threshold. We obtained upper thermal tolerance for all species from a large published database in which estimates were compiled from experimental assays or estimated through phylogenetic imputation of the observed CT_max_ measurements for closely related species (Comte and Olden 2017; Dahlke et al. 2020). When values were not available for our focal species we used values listed from closely related species. Use of phylogenetic imputation is common in fisheries where many species are missing experiential data and represents the best available thermal data at the time of study for these focal species. In our analyses, we used two relevant maxima: (1) the average CT_max_ across all life stages, and (2) the adult CT_max_ (Table S1) (Comte and Olden 2017; Dahlke et al. 2020) to assess the influence of variation in thermal sensitivity across life stages on model predictions. Adult CT_max_ was selected to provide thermal limitations of fisheries relevant (adult‐dominated) populations, while average CT_max_ across life stages was selected to provide more general estimates of thermal vulnerability. Spawning adults and embryos typically have the lowest thermal max values of all life stages. Since we used the average rather than the lowest most constrained values, our models represent the maximum temperatures that could be tolerated by a species and a slightly more constrained estimate rather than a true minimum area of suitability. Model parameters were estimated using Markov chain Monte Carlo (MCMC) simulation through JAGS using *coda* (Plummer et al. 2006), *rjags* (Plummer 2025) and *jagsUI* (Kellner 2024). For all models we ran three MCMC chains in parallel for between 230,000 and 450,000 total iterations depending on the species (Table S2), sampled every 20 iterations with between 200,000 and 400,000 iterations discarded as burn in. We assessed convergence of chains by examining trace plots and Gelman–Rubin statistics for model parameters (all were < 1.1).

Models were validated using k‐fold cross‐validation with 10 folds using *jagshelper* (Tyers 2024). K‐fold is commonly used for model validation of distribution models (Zhang et al. 2024). For each fold, we calculated an omission threshold which indicates the threshold at which the model accurately predicts 90% of known presence locations. This was then used to build a confusion matrix. Using this matrix we calculated “Accuracy,” True Skill Statistic (TSS), Cohen's Kappa, Omission Rate (OR) test value, AUC, and Boyce index. We also extracted Root Mean Squared Error (RMSE) and Mean Absolute Error (MAE) across all folds (Table S1). All metrics helped to assess model accuracy in some capacity. While all utilized metrics are fairly common, there is debate over which provide the best assessment of distribution models. To address this, we opted for redundancy, choosing to calculate both metrics when there was debate over the efficacy of one. The “accuracy” metric assesses the proportion of correctly classified presence and absences using the calculated threshold. Cohen's Kappa was used to assess model agreement between observed and simulated data correcting for how much variation would be expected by chance (Cohen 1960). TSS was included as an alternative to Kappa assessing randomization within accuracy without sample size dependence (Allouche et al. 2006). Omission rate assesses the percentage of presence points that are incorrectly classified as low percent chance of presence and is threshold dependent (Anderson et al. 2003). AUC was included to assess how well the model distinguishes between binary outputs and is typically considered a threshold independent metric for assessing model performance (Hanley and McNeil 1982). We included Boyce index as an alternative to AUC (Hirzel et al. 2006; Zhang et al. 2024), with Boyce index assessing how model predictions differ from random distributions of presences (Boyce et al. 2002). RMSE assesses the average magnitude of error within the model, while MAE assesses how much variance exists between predictions from actual values.

All distribution models were run in RStudio (RStudio Team 2020). Data was processed using *dplyr* (Wickham, François, et al. 2023), *tidyr* (Wickham, Vaughan, and Girlich 2023), and *sf* (Pebesma 2018; Pebesma and Bivand 2023). Mapping and visualizations were run using *ggplot2* (Wickham 2016), *sp* (Bivand 2022; Bivand et al. 2013), *terra* (Hijmans 2025), *ggspatial* (Dunnington 2023), *ggimage* (Yu 2023), *ggpubr (*Kassambara 2025 *), cowplot* (Wilke 2025), and *tidyterra* (Hernangómez 2023). Maps utilized *rnaturalearth* (Massicotte and South 2023) and *rnaturalearthdata* (South et al. 2024) for terrestrial basemaps. MPA boundaries were added to maps using WDPA data (UNEP‐WCMC and IUCN 2025). Confusion matrix was built using *caret* (Kuhn 2008). Boyce index was calculated using *ecospat* (Broennimann et al. 2025). AUC was calculated using *pROC* (Robin et al. 2011).

We fit models to present day temperature conditions that aligned with the time period of occurrence data to define parameters of the plateau model and then used those parameters to generate prediction maps for contemporary conditions, the recent past, and future projected temperatures under the three SSP scenarios. Prediction maps were generated for our entire modeled area, the Caribbean (7°–25° N, 60°–90° W), and for the MAR (Figure S1). We then characterized changes in predicted temperature suitability (i.e., probability of occurrences) by subtracting the “current day” predictions from each future temperature scenario prediction for each pixel across the modeled area. When looking at comparisions of current day to recent past we subtracted “current day” predictions from “recent past” predictions for each pixel. This transformation allows for temperature scenarios to be interpreted with negative values as a loss of relative probability and positive values as an increase in relative probability compared to “current day” estimates.

### 2.2 Analyzing Effects of Changing Climate Suitability on Biomass Trends

We then confronted the estimates of change in SST suitability from pSDMs for each species with long‐term monitoring data from sites across the MAR. Here, we asked whether sites with greater decreases in species‐specific climate suitability are already associated with realized declines in abundances of those species, and conversely, whether sites with greater climate stability tend to better support stable or increasing populations. We analyzed monitoring data maintained by the Healthy Reefs Initiative, a partnership of 70+ organizations working in the MAR. This monitoring network conducts repeated surveys of reef sites using standardized AGRRA methodology at sites along Caribbean coasts of Mexico, Belize, Guatemala, and Honduras (NCRMN 2025). At each site and during each sampling occasion, dive teams count reef fish along a minimum of 10 replicated 30 × 2‐m belt transects, each seperated by at least 10 m and haphazardaly placed. Observed fish were identified to species and their total length (TL) was visually estimated and binned into size classes according to the AGRRA protocol (Lang et al. 2010). Biomass was then estimated using TL and allometric equations. Aallometric length‐weight parameter data was collated from FishBase (Froese and Pauly 2020). To analyze spatial variation in changes in biomass over time, we only included surveyed sites for which transects had been sampled in three or more years from 2005 to 2018, resulting in 139 sites retained for analysis across the MAR. At each site, we extracted mean estimated probability of occurence from the plateau SDMs for the recent past and current day conditions for each species within a 5‐km buffer, calculating the mean values across cells within the buffer. We also calculated the change in suitability from past to present. To account for other known drivers of fish abundances in the analyses, we also extracted Human foortprint index at each site to quantify level of coastal development (Venter et al. 2016) and estimated fishing effort form a coarse gridded global dataset (Rousseau et al. 2024). Finally, we included live coral cover from the AGRRA transect data at each site as a known predictor of fish biomass in this and other regions (Canty et al. 2024). We assessed correlations among these predictors, which were weakly to moderately correlated (*r* = −0.004 to −0.567).

To examine whether change in climate suitability, from recent past to current day conditions, explains spatial variation in biomass trends of each species, we fit a Bayesian multilevel model (Gelman and Hill 2006) (see JAGS code in Supporting Information) with change in climate suitability and the other spatial drivers as predictors variables. We fit a model with a Gamma probability distribution due to the positive and right‐skewed distribution of the biomass response variable. We added a constant value of 1 to each biomass observation in the dataset to accomodate the log link within the likelihood function, such that observations with zero biomass equal zero following log transformation. We fit the model with varying intercepts and slopes for subregions (groups of sites within countries) and sites. In one level of the model, the varying slopes represented the estimated change in biomass across years at regional and site scales for each of the focal species, indexed in the model. In a second level of the model, we estimated the effects of changes in climate suitability and other spatial covariates for each species as predictors of variation among sites in biomass trends (i.e., a linear submodel of the effects of suitability on the varying slope parameters for each species). For these coeffiecients, we used vague, normal priors. The multilevel model allowed us to simultaneously generate estimates of biomass trends for each species and site while integrating uncertainty in trend estimates into estimates of the effects of covariates on biomass trends across the MAR for each species.

Prior to the analysis, we centered and scaled all predictor variables. We fit the model in JAGS using the *jagsUI* package (Kellner 2024) to interface with R. We ran four MCMC chains in parallel for a total of 20,000 iterations, sampled every 20 iterations, with 10,000 discarded as burn‐in. We examined traceplots and Gelman‐Rubin statistics to assess mixing and convergence for each parameter.

## 3 Results

### 3.1 Projected Changes in Temperature Suitability

The pSDMs were validated using several metrics (Table S1). Boyce index k‐fold average values ranged from 0.54 to 0.77 across all species and models indicating models were better than random at predicting suitable habitat. AUC average k‐fold values ranged between 0.88 and 0.94 indicating better than random model discrimination without concern of model overfitting. RMSEs from our K‐fold cross validation were between 0.35 and 0.46, indicating low error within our models. Other performed validation metrics including Cohen's Kappa, MAE, Accuracy, OR Test, and TSS confirmed good model fit (Table S1). Individual values for all folds can be found in the supplement (Table S3).

Physiology informed models of finfish species identified areas of declining climate suitability as well as areas of stability with variation in severity of declines both spatially and across species by the end of century throughout the Caribbean (Figure 1). Projected declines in climate suitability were generally more severe when using the average CT_max_ across life stages as a prior within pSDMs than when using adult CT_max_ (Figures 1 and S5). When looking at suitability values of all species across the Caribbean, average life stage CT_max_ models had a mean relative probability of occurrence of 75% under current day conditions. This value declined to 30% under SSP4.5 (change of −45%), 22% under SSP6.0 (change of −53%), and 9% under SSP8.5 (change of −66%). For the same region, adult CT_max_ models had an average of 73% relative probability of occurrence at current day, 52% under SSP4.5 conditions (change of −21%), 47% under SSP6 (change of −26%), and 30% under SSP8.5 (change of −43%).

**FIGURE 1:**
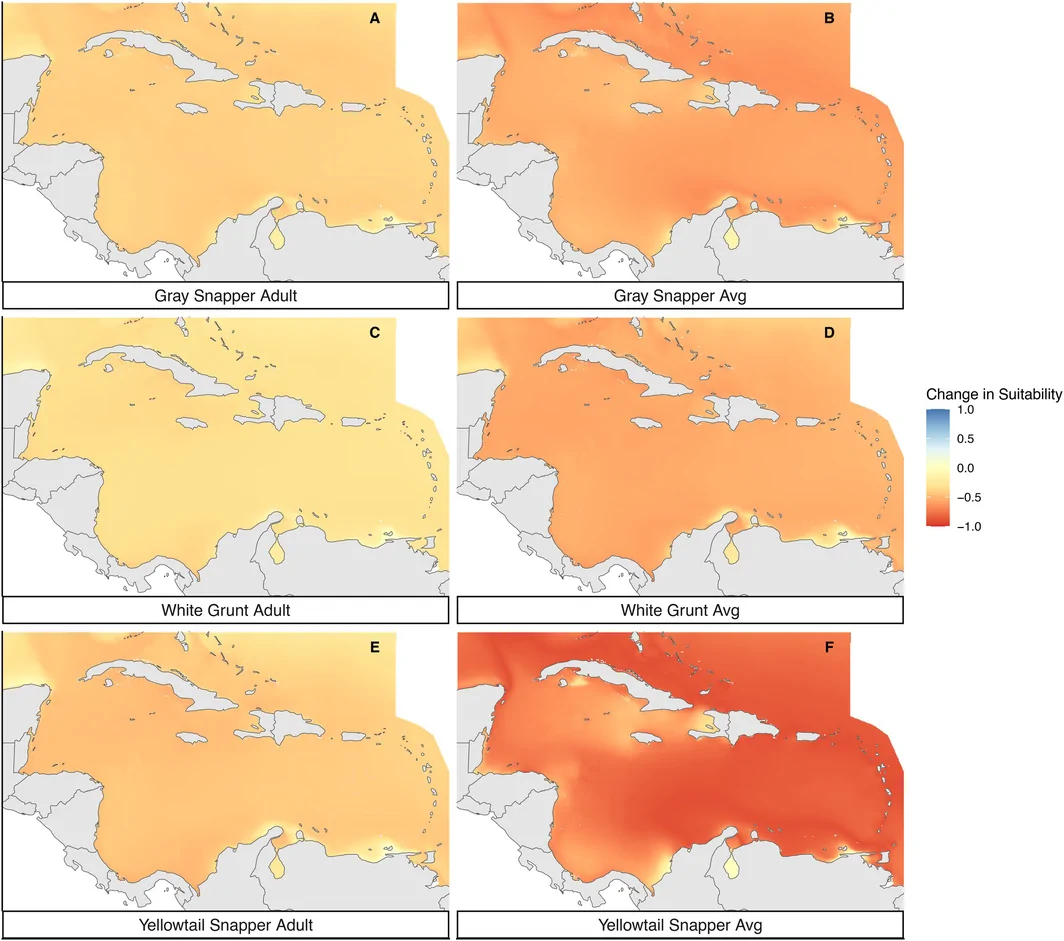
Change in projected habitat suitability from current day to SSP5‐8.5 at end of century for three finfish species. Left plots represent Adult CT_max_, while right plots represent average CT_max_. Cool colors indicate an increase in probability of occurrence while warm colors indicate a decrease in probability of occurrence, with deeper red shades indicating more sever declines. Projection maps for additional species can be found in Figure S9.

When zooming into the MAR, there are clear trends of suitability declines across the eastern Yucatan peninsula in Quintana Roo, while areas of northern Yucatan, Yucatan State, have more stable suitability values (Figures 2 and S6). For projections with the adult CT_max_, average probability of occurrence across species dropped from 70% at current day to 41% under SSP6 and 25% under SSP8.5. These projections show more severe declines when modeled with the average CT_max_ when compared to the adult CT_max_, aligning with Caribbean regional trends (Figure 3). Projections using the average CT_max_ dropped from 71% relative probability of occurrence under current day to 14% under SSP6, and only 6% under SSP8.5. Patterns are similar across species, with the largest declines seen in barracuda and yellowtail snapper. Barracuda projections using adult CT_max_ dropped from 71% under current day conditions to 33% under SSP6 and 16% under SSP8.5. White grunt appeared to do the best under high warming scenarios in the MAR showing the most moderate change in suitability with estimates dropping from 75% under current day adult CT_max_, to 55% under SSP6 and 42% under SSP8.5.

**FIGURE 2:**
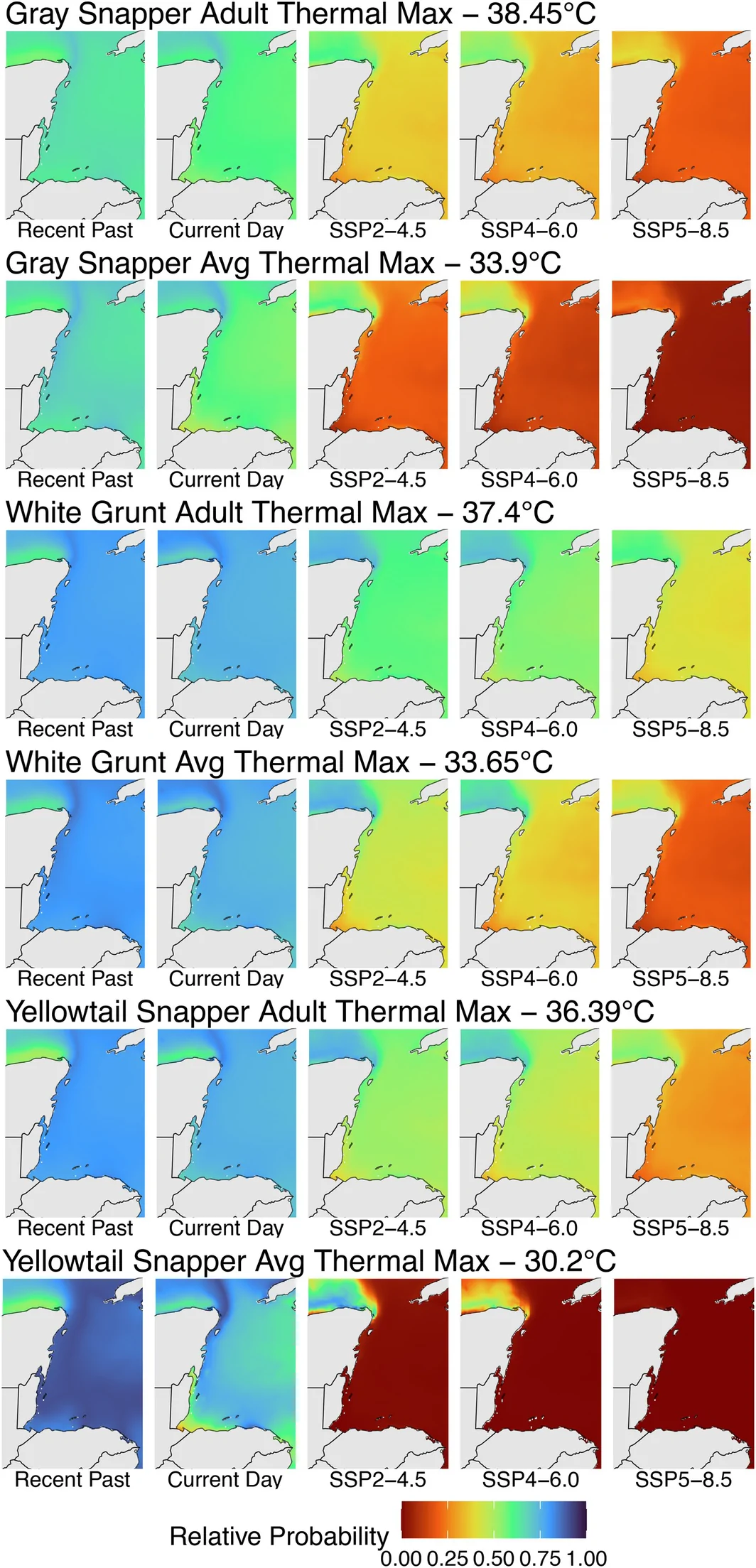
Habitat Suitability Maps of three modeled finfish species, selected to highlight a range of responses under climate change scenarios for end of century (2100). Outputs shown for both average CT_max_ and adult CT_max_. Cool colors indicate higher probability of occurrence while warm colors indicate lower probability of occurrence.

**FIGURE 3:**
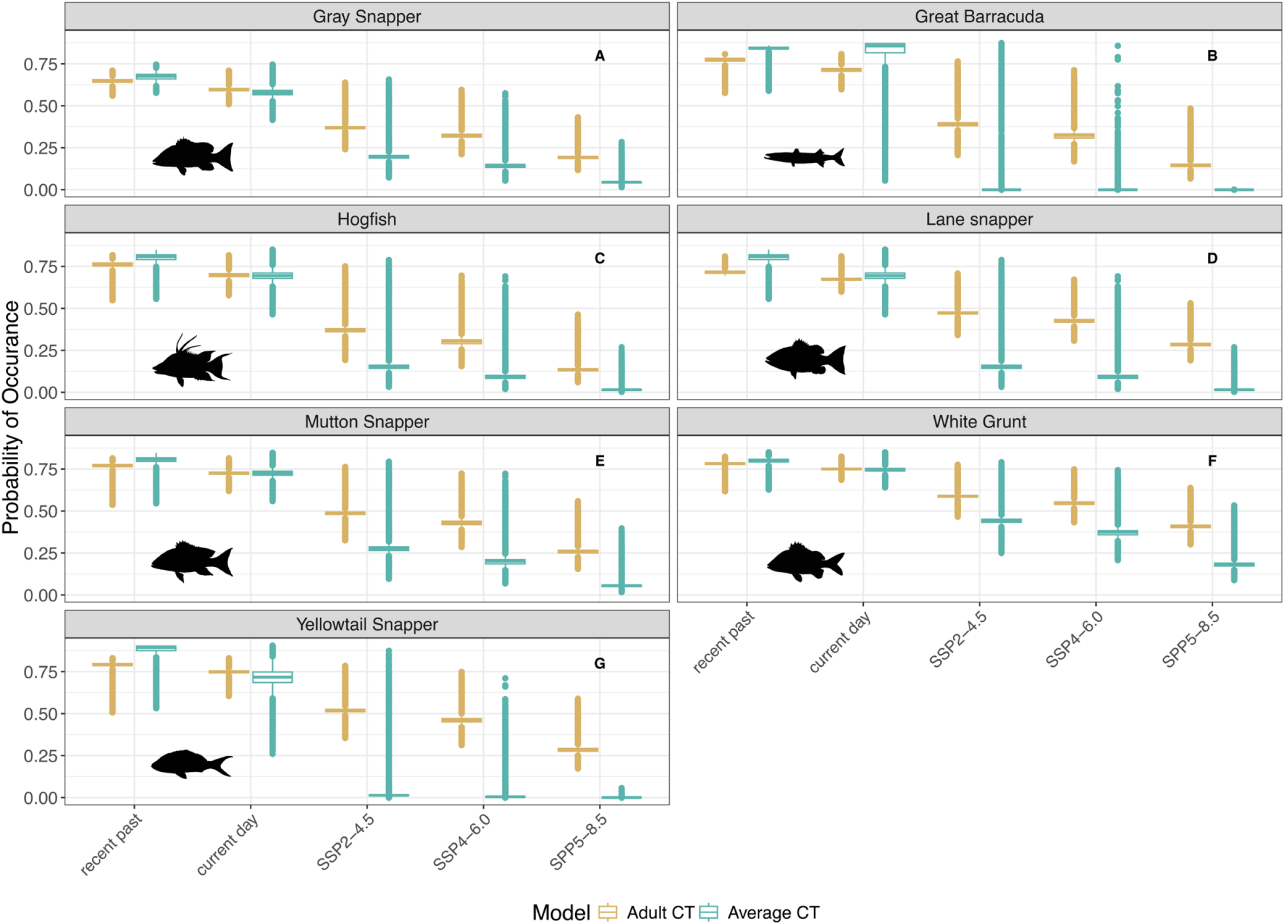
Boxplots of probability of occurrence values within the Mesoamerican Reef region for each modeling scenario. Fish suitability declined between current day and future climate scenarios for panels with more severe declines in Average CT models than Adult CT models. Orange boxes represent Adult CT_max_ models, while teal boxes represent Average CT_max_ models. Boxes represent the first and third quartile, with median indicated by the horizontal line. Whiskers show the data range extending from the upper hinge to the largest value up to 1.5 times the inter‐quartile range (IQR) or the smallest value from the lower hinge at most 1.5 times IQR, with dots representing outliers.

### 3.2 The Effects of Changing Temperature Suitability on Biomass Trends in the MAR

Change in climate suitability across the MAR was positively associated with biomass trends for some species, indicating declines in biomass for those species are occurring in areas with greater decreases in climate suitability in the recent past (Figures 4 and S7, and Table S4). The overall mean effect of climate suitability across species was only weakly positive due to variation in effects among species (mean *β* = 0.179, 95% Bayesian Credible Interval (BCI) = [−0.312, 0.638]). However, there was statistical support for the effect of changing climate suitability (Bayesian Credible Intervals exclude 0) for barracuda (mean *β* = 0.42, 95% BCI = [0.12, 0.71]), mutton snapper (mean *β* = 0.35, 95% BCI = [0.003, 0.70]), and white grunt (mean *β* = 0.33, 95% BCI = [0.08, 0.59]), all of which exhibited greater declines, on average, in areas with greater decreases in climate suitability (Figures 4 and S7). The direction of effect of suitability change on biomass was similar for yellowtail snapper, but was not significant. Lane snapper, hogfish, and gray snapper, in contrast, exhibited no discernible (or slightly negative in the case of hogfish) responses to spatial variation in suitability change. Notably, lane snapper, hogfish, and gray snapper have relatively high CT_max_ and were also less abundant in transect surveys, on average, than the other four species (Table S4). In the case of hogfish, the biomass of local populations was significantly declining throughout the MAR (mean *β* = −0.43, BCI = [−0.73, −0.07]). There was a significant positive effect of live coral cover on biomass trends for gray snapper, mutton snapper, and white grunt (Table S4). The other covariates did not have significant effects. Taken together, these results indicate that some species are already declining in areas where climate suitability has decreased in recent decades, and the effects of climate change on subsistence fisheries are species dependent.

**FIGURE 4:**
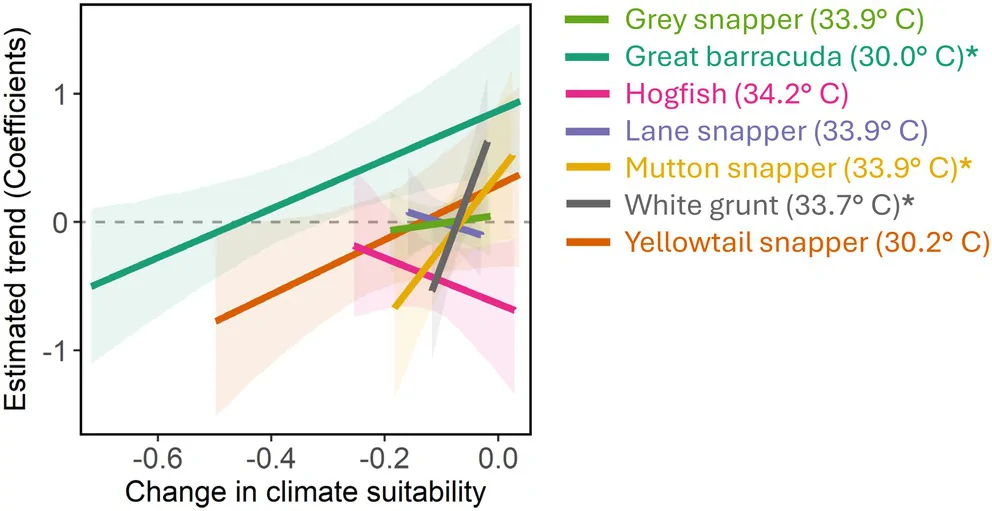
Trends in fish biomass tend to be more stable or increase in areas where climate suitability has been more stable in the past 20 years within the Mesoamerican Reef region. We estimated trends in biomass of species at 139 sites that were monitored with transect surveys from 2005 to 2018 and the effects of change in climate suitability using a Bayesian multilevel model. We estimated change in climate suitability using physiological species distribution models for each species. The thermal tolerance (Avg CT_max_) of each species is provided in parentheses. Asterisks indicate statistical support for associations between biomass trends and change in climate suitability (i.e., 95% Bayesian Credible Intervals for slope parameters exclude 0). Shaded areas represent 95% credible bands. Site‐level trend estimates are shown in Figure S7.

## 4 Discussion

Ongoing declines and projections of decreasing climate suitability across the region could have detrimental effects for coastal communities dependent on finfish species as one of their primary forms of subsistence. As of 2016, one third of countries studied could not meet their subsistence goals based on fisheries catch rates (Canty and Deichmann 2022). While changes in availability from overfishing have already led to fisheries adaptation, further declines in catch could leave thousands of coastal communities throughout the region food insecure and in need of alternative food sources. In the MAR alone, there are 1.5 million people who live within 5 km of the coast (Archibald et al., n.d.; Bondarenko et al. 2025). Trends in biomass and climate suitability across the studied species indicate potential for substantial impacts on small‐scale subsistence and commercial fisheries, thereby affecting food security through reduced subsistence catch and income in coastal fishing communities (Lam et al. 2012; Nowakowski et al. 2023; Sarre et al. 2024). Additionally, shifts in species ranges create accessibility challenges for coastal subsistence fishers who have limited access to boats and other equipment needed to track fish population movement, especially offshore (Bell et al. 2013). These potential impacts of climate‐driven shifts in fish distributions highlight the need for incorporating projected responses of key species, including up and coming species, into spatial planning for marine resource management.

Physiology‐informed SDMs are known to generate more accurate predictions of species distributions than traditional SDMs (Feng et al. 2020) due to their ability to reduce over‐projection beyond known physiological limits. This aspect becomes pivotal when trying to ensure long‐term resource availability under changing climate conditions. Our physiology‐informed models generated a wider response curve for most species indicating a larger thermal niche than predicted by non‐physiology‐informed models (Figure S8). This is likely due to the intentional inclusion of the entire niche space under the response curve, and the incorporation of occurrences at high temperatures that are sparsely sampled but under the theoretical species' thermal max. This integration of physiological thresholds into distribution models helps us to understand spatial variation in species' thermal responses and add biological realism to projections of climate suitability into the future.

While outputs of physiologically informed SDMs indicated widespread decreases in temperature suitability throughout much of the Caribbean, they also highlighted a few areas of SST stability, where there was minimal projected change in suitability compared to current day. These areas include the northern Yucatan peninsula along Yucatan State and Venezuela (Figure 1). These areas of projected stability provide insights into potential locations of thermal refuge and climate resilience. Overlaying current MPAs onto modeled outputs helps to identify areas that are currently vulnerable to fishing pressure and could be good candidates for the expansion or implementation of new MPAs (Figure S9). When looking across the region, the distribution of changes in climate suitability within the current MPA network largely tracked the background distribution of suitability trends for the entire region (i.e., are randomly distributed with respect to climate; Figure S10). A notable difference in these distributions is that MPAs do not currently cover areas in the region that have the greatest climate stability (i.e., potential refugia). This result indicates that MPAs were not selected intentionally to include areas of thermal refugia, and reveals an important gap in resilience focused management and MPA expansion that could be easily filled by planning of future climate resilient MPAs. Integration of predicted climate refugia into spatial planning of MPA network expansion could bolster fish populations via both reduced fishing pressure and ensuring preservation of areas with low thermal stress, thereby increasing resilience of key species (Arafeh‐Dalmau et al. 2023; Wilson et al. 2020).

Additionally, looking at the average change in suitability across species within currently established MPAs can provide managers with a metric of potential relative thermal stress and aid in planning for climate change (Figure S11). Extracting these values for our models revealed San Pedro Underwater Archaeological Preserve in Florida as having the highest expected decline in suitability across species and life stages; around −0.75. Other MPAs, like Utilidad Pública y de Interés Turístico Recreacional El Castillo de Araya in Venezuela, are expected to see more stability in their fish populations with suitability values increasing around 0.15. While these estimates are not guarantees that fisheries will decline, they do provide general trends for how severe thermal stress could influence fish distributions and communities in the future.

The severity of projected declines in climate suitability varied based on the realized climate niche and the upper thermal limits of each species as well as life stages. The pSDMs predicted more severe declines in suitability when projections were governed by the average CT_max_ across life stages than adult CT_max_ (Figures 2 and S6), due to the narrower thermal range of spawning adults and embryos (Dahlke et al. 2020). Greater vulnerability of these life stages could impact recruitment and development success of larval and juvenile fish. As a result, models informed only by adult thermal tolerances could overestimate the extent of climate refugia. With nursery and spawning grounds particularly at risk of thermal stress, the intentional inclusion of areas within marine reserves and management zones that can act as climate refugia could increase the effectiveness of conservation areas across life stages and may facilitate fisheries recovery (Arafeh‐Dalmau et al. 2023; Krueck et al. 2018).

In addition to variation across life stages, we observed species level variation in the severity of thermal response. We focused on a small subset of the reef fish community in the MAR and modeled species CT_max_ values that are not representative of the full range of temperature sensitivity for species in the region. There are species with higher and lower thermal tolerances than those in this study, with species represented here exhibiting an intermediate range of thermal tolerance. The focal species in this study with the most severe projected declines typically had lower CT_max_ values. Barracuda and yellowtail snapper, with lower average life stage CT_max_ values, had more severe projected declines at high warming scenarios than white grunt or lane snapper, species with greater average life stage CT_max_ (Figures 3 and S5). While some species fit this more linear relationship, other species' responses were less intuitive. Hogfish, which also has a relatively high average life stage CT_max_, had higher projected declines than other species with a similar thermal maxima. These deviations from a linear relationship could be due to a variety of reasons, including that within the model it is not the upper thermal limit alone determining suitability but the interaction of the upper limit on the logistic regression of occurrence data. It is likely that the broader fish community will experience even greater species level variation in severity of responses, with potential for some species to have more positive responses to warming, while the community as a whole simultaneously experiences an overarching decline in suitability.

Within the MAR region, we saw similar declines in suitability as we did across the broader Caribbean. We then integrated changes in climate suitability with long term fish monitoring data at sites across the MAR, finding that declines in biomass have already occurred in areas with decreasing climate suitability for some species within this region (Figure 4). These trends in long‐term monitoring data help to ground‐truth the pSDM model outputs, providing real world evidence of theoretical model outputs. That spatial variation in projected declines in suitability is associated with observed fish biomass declines, underscoring the urgency of addressing ocean warming and the necessity of climate‐resilient management.

While temperature is likely one dominant driver of the relationship between long‐term monitoring data and projected suitability, multiple stressors are present in the region and likely contributing to declines with interactions among climate change, coastal development, fishing pressure, and management (Canty et al. 2024). Although our results support the expectations that spatial variation in climate suitability covaries with rates of fish declines across sites, the analysis is correlative in nature and we cannot rule out other factors that may have driven these trends. Overfishing, in particular, is strongly related to individual body size, leading to size selection of larger individuals from a population, while smaller individuals reduce body size in response to warming (Genner et al. 2010), leading to reductions in body size across the size spectrum. However, high‐resolution data on spatial variation in fishing pressure are not available for the region.

Protection of climate refugia via climate resilient MPA networks can help address many of these compounding stressors, while increasing connectivity to facilitate local population persistence and range shifts. Observed declines in biomass may be early signs of impending range shifts, with up to 50% of climate‐related biomass reductions being attributed to changes in range dynamics (Cheung et al. 2013). These range shifts have the potential to cause major changes in the availability and accessibility of preferred species for subsistence fishers. Successful integration of climate vulnerability assessments into marine spatial planning has the potential to mitigate the impacts of climate change on food security in coastal populations reliant on subsistence fishing.

Over the last 30 years, there have been significant efforts across the MAR to increase climate resilience and bolster marine reserves but there are still disconnects between scientific evidence and practice (Archibald et al., n.d.). The Tulum Declaration (1997), a foundational environmental agreement among MAR countries, promotes transboundary cooperation on marine conservation issues in the region (Alvarez and Wong 2015). This cooperation is particularly important when thinking about planning efforts for species undergoing range shifts and that hold value for multiple nations. The percentage of territorial seas within MPAs across the MAR has increased over time with the adoption of area‐based targets under the Global Biodiversity Framework and Strategic Plan for Biodiversity. However, the vast majority of these MPAs are not fully protected and allow for some level of fishing (Nowakowski et al. 2023). Additionally, illegal fishing has continued to rise in response to declining fish stocks and reduced catch (Rivera et al. 2025), highlighting the vulnerability of fish populations in this region that will only increase as oceans continue to warm. The small percentage of fully protected areas in the region has been successful in bolstering populations of key subsistence species including snapper (Healthy Reefs 2024; Nowakowski et al. 2023). Increasing enforcement of established MPAs, in addition to expansion of the MPA network to include critical climate refugia, would ensure that international protection goals like the Global Biodiversity Framework 30 × 30 targets are not only met, but also that protected areas are climate resilient and effective in maintaining fish populations and increasing co‐benefits to local communities (Nowakowski et al. 2023).

Despite ongoing climate‐related declines of some species and projected decline in suitability for all species, reduced suitability could manifest in different ways. Species could potentially acclimate to warming waters and more extreme heat events given sufficient individual plasticity in thermal tolerance (Witman et al. 2023). Local adaptation could also occur if heat induced die‐offs lead to population level selection for warm‐adapted individuals with higher thermal tolerance resulting in a shift in the thermal niche (Gilbert and Miles 2017). However, much research suggests that CT_max_ tends to be highly conserved with limited acclimation and adaptation potential (Desforges et al. 2023; Madeira et al. 2012), indicating that other outcomes are more likely. Across large scales, we expect changes in occurrence to lead to range shifts, both across latitudes and depths, as species attempt to track their current thermal niche (Pinsky et al. 2020). How these changes play out across geographic and temporal scales will be highly localized and depends on the interaction of frequency and scale of warming, thermal plasticity within the population, and accessibility of thermal refugia as well as on each species' natural history. For example, species could become locally rarer in habitats with higher temperatures like nearshore habitats or locations with less tidal flow. They could start to favor areas that allow for consistently cooler water temperatures and less thermal stress. Furthermore, it is possible that fishers will switch their efforts to other more abundant species. However, this and other analyses (Canty et al. 2024; Magel et al. 2020) suggest large scale shifts in biomass and demographic structure are driven in part by climate change in the region, affecting the broader fish community and food web. Our results highlight the potential for large‐scale reductions in climate suitability within the study region for all modeled focal species under all climate change conditions with variance in severity of declines among species. The pSDMs also revealed key differences in suitability based on adult thermal max with average thermal max across all life stages producing greater reductions in suitability than models that only accounted for adult thermal max—providing insights into climate refugia for different life stages. Additionally, climate vulnerability from pSDMs explained spatial variation in the MAR region's long‐term monitoring data helping us to understand ongoing population trends in the region. Taken together, these results underscore the need to integrate climate vulnerability assessments into marine spatial planning. By identifying areas of climate refuge, resource managers and policy makers can improve prioritization of conservation areas to support food security and ensure protected area networks are climate resilient for 2030 and beyond.

## Author Contributions

**Mary R. Cortese:** conceptualization, methodology, validation, visualization, writing – review and editing, writing – original draft, formal analysis, data curation, investigation. **Steven W. J. Canty:** conceptualization, writing – review and editing, supervision, funding acquisition. **Sara M. Melo‐Merino:** writing – review and editing, methodology, validation. **A. Justin Nowakowski:** conceptualization, validation, methodology, supervision, writing – review and editing, funding acquisition.

## Funding

M.R.C. and S.M.M.‐M. received funding from the Smithsonian Institution's Life on a Sustainable Planet initiative, and S.W.J.C. received funding from the Summit Foundation.

## Conflicts of Interest

The authors declare no conflicts of interest.

## Supporting information


**Table S1:** Model diagnostics for all species. Values are the average across all k‐folds. Outputs for all folds individually can be found in the supplement (Table S3).
**Tabel S2:** Number of total model iterations and burn‐in for each species and model type. Numbers were consistent across CT_max_ for all physiologically informed models.
**Table S3:** Model diagnostics for all species across all K‐folds. Calculated metrics include “Accuracy,” True Skill Statistic (TSS), Cohen's Kappa, Omission Rate (OR) test value, AUC and Boyce index. The “Threshold” is the omission threshold at which the model accurately predicts 90% of known presence locations. This was then used to build a confusion matrix and calculate the listed values.
**Table S4:** Mean estimates and lower (LCI) and upper (UCI) 95% Bayesian credible intervals (BCIs) from a multilevel model examining the effects of changing climate suitability and other know drivers of fish abundance on biomass of each species across 139 sites throughout the MAR. Intercepts represent mean biomass trends across the monitoring period and across all monitored sites. Climate suitability effects are the coefficients representing change in biomass trends across the monitoring period with variation in changing climate suitability across sites surveyed in the MAR. Rhat is the Gelman–Rubin statistic and Sig indicates estimates where BCIs exclude 0.
**Figure S1:** Map of modeled region and Mesoamerican Reef subregion.
**Figure S2:** Comparison between current day mean monthly mean SST layers from BioOracle and Coral Reef watch. Summaries show kernel density plot and boxplot of the two sst layers. Boxes represent the first and third quartile, with median indicated by the horizontal line. Whiskers show the data range extending from the upper hinge to the largest value up to 1.5 times the inter‐quartile range (IQR) or the smallest value from the lower hinge at most 1.5 times IQR, with dots representing outliers.
**Figure S3:** Density plots of SST data layers. Three data layers are shown including the Bio‐Oracle (BO) mean monthly mean SST data for current day and three future scenarios, current day coral reef watch (CRW) mean monthly max SST, and adjusted future layers. We adjusted future scenarios to projections of mean monthly maximum by adding the difference between current BO data and each BO future mean monthly mean SST layer to the current day CRW mean monthly max data.
**Figure S4:** Theoretical response curve demonstrating the three segmented curves, along with the MTP and CTmax constraints on the right segment. Original model design from Feng et al. (2020).
**Figure S5:** Boxplots of probability values within the Caribbean (60–90 W, 7–25 N). Fish suitability declined between current day and future scenarios for panels with more severe declines in Average CT models than Adult CT models. Orange boxes represent Adult CTmax models, while teal boxes represent Average CTmax models. Boxes represent the first and third quartile, with median indicated by the horizontal line. Whiskers show the data range extending from the upper hinge to the largest value up to 1.5 times the inter‐quartile range (IQR) or the smallest value from the lower hinge at most 1.5 times IQR, with dots representing outliers.
**Figure S6:** Habitat suitability maps for four other species, focused on the Mesoamerican Reef. Adult and Avg CTmax. Outputs shown for both average CTmax and adult CTmax. Cool colors indicate higher probability of occurrence while warm colors indicate lower probability of occurrence. Climate projections shown for end of century (2100).
**Figure S7:** Trends in fish biomass tend to be more stable or increase in areas where climate suitability has been more stable in the recent past within the Mesoamerican Reef region, in particular for Great barracuda (Sphyraena barracuda), mutton snapper (Lutjanus analis), and white grunt (Haemulon plumierii). We estimated trends in biomass of species at 139 sites that were monitored with transect surveys from 2005 to 2018 and the effects of change in climate suitability using a Bayesian multilevel model. We estimated change in climate suitability using physiological species distribution models for each species. Solid triangles indicate significant negative or positive site‐level trends in biomass, and transparent circles indicate non‐significant site trends. Error bars represent 95% Bayesian credible intervals for trend estimates at each site.
**Figure S8:** Response curves of all seven species to temperature. Black line represents response of the physiology informed model. Orange line represents response of the uninformed model. Models run with average CTmax are shown in A panels, while adult CTmax is shown in B panels.
**Figure S9:** Change in probability of occurrence from current day to RCP 8.5 end of century. Warm colors indicate a larger reduction in thermal suitability while cool colors represent an increase in thermal suitability. Outputs are included for all seven finfish species and both average and adult CTmax. Current marine protected areas are included as white overlayed polygons.
**Figure S10:** Change in suitability relative to conservation status. Trends are similar across models with MPAs suitability change largely matching the background distribution. Histograms show the MPAs have similar change in suitability estimates as the surrounding areas indicating that MPAs were not selected for their climate resilience. Green bars represent values within an MPA, while blue bars represent all other areas within the Caribbean.
**Figure S11:** Change in probability of occurrence from current day to RCP 8.5 end of century. Warm colors indicate a larger reduction in thermal suitability while cool colors represent an increase in thermal suitability. Panel A represents adult CTmax models while panel B represents average CTmax models Change maps for each species were extracted from with the MPA boundary, averaged across species and each pixel to give a singular average change value for each MPA.
**Supplemental Code 1:** JAGS code used to model trends in biomass as a function of change in temperature suitability from SDMs.

## Data Availability

Data and code for the distribution models are available on FigShare, DOI: 10.6084/m9.figshare.30935696. Data using in biomass trend models is available by request from the Atlantic and Gulf Rapid Reef Assessment database at https://agrra‐data‐explorer‐oref.hub.arcgis.com/ and Healthy Reefs Initiative Eco‐Audits at www.healthyreefs.org.
