# Supplementary material for: Thermal Vulnerability of Reef Fish and Implications for Subsistence Fisheries Throughout the Caribbean
Source: Glob Chang Biol. 2026 Oct 6;32(10):e71118. doi: 10.1111/gcb.71118 (PMC13643191; doi:10.1111/gcb.71118)
Supplement: Supplementary file 1 [file GCB-32-e71118-s001.docx]

Thermal vulnerability of reef fish and implications for subsistence fisheries throughout the Caribbean

Supplement:

Table S1: Model diagnostics for all species. Values are the average across all k-folds. Outputs for all folds individually can be found in the supplement (Table S3).

Tabel S2: Number of total model iterations and burn-in for each species and model type. Numbers were consistent across CT_max_ for all physiologically informed models.

Table S3: Model diagnostics for all species across all K-folds. Calculated metrics include ‘Accuracy’, True Skill Statistic (TSS), Cohen’s Kappa, Omission Rate (OR) test value, AUC, and Boyce index. The ‘Threshold’ is the omission threshold at which the model accurately predicts 90% of known presence locations. This was then used to build a confusion matrix and calculate the listed values.

Table S4: Mean estimates and lower (LCI) and upper (UCI) 95% Bayesian credible intervals (BCIs) from a multilevel model examining the effects of changing climate suitability and other know drivers of fish abundance on biomass of each species across 139 sites throughout the MAR. Intercepts represent mean biomass trends across the monitoring period and across all monitored sites. Climate suitability effects are the coefficients representing change in biomass trends across the monitoring period with variation in changing climate suitability across sites surveyed in the MAR. Rhat is the Gelman-Rubin statistic and Sig indicates estimates where BCIs exclude 0.

| Species | Variable | Mean est. | 95% LCI | 95% UCI | Rhat | Sig. |
| --- | --- | --- | --- | --- | --- | --- |
| Sphyraena barracuda | Intercept (mean trend) | 0.225 | -0.057 | 0.525 | 1.009 |  |
| Sphyraena barracuda | Live coral cover | 0.232 | -0.030 | 0.496 | 1.001 |  |
| Sphyraena barracuda | Human footprint index | 0.053 | -0.263 | 0.421 | 1.002 |  |
| Sphyraena barracuda | Fishing pressure | 0.051 | -0.229 | 0.319 | 1.009 |  |
| Sphyraena barracuda | Climate suitability change | 0.415 | 0.124 | 0.708 | 1.001 | * |
| Ocyurus chrysurus | Intercept (mean trend) | -0.198 | -0.554 | 0.158 | 1.009 |  |
| Ocyurus chrysurus | Live coral cover | 0.104 | -0.128 | 0.341 | 1.001 |  |
| Ocyurus chrysurus | Human footprint index | -0.162 | -0.544 | 0.210 | 1.006 |  |
| Ocyurus chrysurus | Fishing pressure | 0.300 | -0.023 | 0.661 | 1.006 |  |
| Ocyurus chrysurus | Climate suitability change | 0.326 | -0.044 | 0.695 | 1.008 |  |
| Lutjanus synagris | Intercept (mean trend) | -0.005 | -0.169 | 0.161 | 1.003 |  |
| Lutjanus synagris | Live coral cover | 0.022 | -0.133 | 0.186 | 1.001 |  |
| Lutjanus synagris | Human footprint index | 0.077 | -0.109 | 0.270 | 1.000 |  |
| Lutjanus synagris | Fishing pressure | -0.075 | -0.241 | 0.100 | 1.000 |  |
| Lutjanus synagris | Climate suitability change | -0.050 | -0.241 | 0.134 | 1.001 |  |
| Lachnolaimus maximus | Intercept (mean trend) | -0.426 | -0.725 | -0.072 | 1.032 | * |
| Lachnolaimus maximus | Live coral cover | -0.051 | -0.284 | 0.178 | 1.000 |  |
| Lachnolaimus maximus | Human footprint index | -0.141 | -0.465 | 0.203 | 1.010 |  |
| Lachnolaimus maximus | Fishing pressure | -0.128 | -0.439 | 0.175 | 1.005 |  |
| Lachnolaimus maximus | Climate suitability change | -0.151 | -0.495 | 0.159 | 1.013 |  |
| Lutjanus griseus | Intercept (mean trend) | -0.009 | -0.148 | 0.128 | 1.005 |  |
| Lutjanus griseus | Live coral cover | 0.155 | 0.021 | 0.293 | 1.001 | * |
| Lutjanus griseus | Human footprint index | 0.025 | -0.145 | 0.208 | 1.002 |  |
| Lutjanus griseus | Fishing pressure | 0.007 | -0.145 | 0.159 | 1.000 |  |
| Lutjanus griseus | Climate suitability change | 0.032 | -0.133 | 0.216 | 1.000 |  |
| Lutjanus analis | Intercept (mean trend) | -0.079 | -0.400 | 0.264 | 1.007 |  |
| Lutjanus analis | Live coral cover | 0.330 | 0.074 | 0.595 | 1.001 | * |
| Lutjanus analis | Human footprint index | -0.237 | -0.574 | 0.101 | 1.003 |  |
| Lutjanus analis | Fishing pressure | 0.047 | -0.268 | 0.389 | 1.004 |  |
| Lutjanus analis | Climate suitability change | 0.347 | 0.003 | 0.701 | 1.007 | * |
| Haemulon plumierii | Intercept (mean trend) | 0.039 | -0.213 | 0.296 | 1.010 |  |
| Haemulon plumierii | Live coral cover | 0.218 | 0.015 | 0.413 | 1.000 | * |
| Haemulon plumierii | Human footprint index | 0.041 | -0.242 | 0.308 | 1.004 |  |
| Haemulon plumierii | Fishing pressure | -0.102 | -0.369 | 0.133 | 1.005 |  |
| Haemulon plumierii | Climate suitability change | 0.334 | 0.075 | 0.594 | 1.000 | * |

Figure S1: Map of modeled region and Mesoamerican Reef subregion.

Fig S2: Comparison between current day mean monthly mean SST layers from BioOracle and Coral Reef watch. Summaries show kernel density plot and boxplot of the two sst layers. Boxes represent the first and third quartile, with median indicated by the horizontal line. Whiskers show the data range extending from the upper hinge to the largest value up to 1.5 times the inter-quartile range (IQR) or the smallest value from the lower hinge at most 1.5 times IQR, with dots representing outliers.

Fig S3: Density plots of SST data layers. Three data layers are shown including the Bio-Oracle (BO) mean monthly mean SST data for current day and three future scenarios, current day coral reef watch (CRW) mean monthly max SST, and adjusted future layers. We adjusted future scenarios to projections of mean monthly maximum by adding the difference between current BO data and each BO future mean monthly mean SST layer to the current day CRW mean monthly max data.

Fig S4: Theoretical response curve demonstrating the three segmented curves, along with the MTP and CTmax constraints on the right segment. Original model design from Feng, 2020.

Figure S5: Boxplots of probability values within the Caribbean (60-90 W, 7-25 N). Fish suitability declined between current day and future scenarios for panels with more severe declines in Average CT models than Adult CT models. Orange boxes represent Adult CTmax models, while teal boxes represent Average CTmax models. Boxes represent the first and third quartile, with median indicated by the horizontal line. Whiskers show the data range extending from the upper hinge to the largest value up to 1.5 times the inter-quartile range (IQR) or the smallest value from the lower hinge at most 1.5 times IQR, with dots representing outliers.

Figure S6: Habitat suitability maps for four other species, focused on the Mesoamerican Reef. Adult and Avg CT_max_. Outputs shown for both average CT_max_ and adult CT_max_. Cool colors indicate higher probability of occurrence while warm colors indicate lower probability of occurrence. Climate projections shown for end of century (2100).

Figure S7: Trends in fish biomass tend to be more stable or increase in areas where climate suitability has been more stable in the recent past within the Mesoamerican Reef region, in particular for Great barracuda *(Sphyraena barracuda),* mutton snapper (*Lutjanus analis*), and white grunt (*Haemulon plumierii)*. We estimated trends in biomass of species at 139 sites that were monitored with transect surveys from 2005-2018 and the effects of change in climate suitability using a Bayesian multilevel model. We estimated change in climate suitability using physiological species distribution models for each species. Solid triangles indicate significant negative or positive site-level trends in biomass, and transparent circles indicate non-significant site trends. Error bars represent 95% Bayesian credible intervals for trend estimates at each site.

Figure S8: Response curves of all seven species to temperature. Black line represents response of the physiology informed model. Orange line represents response of the uninformed model. Models run with average CT_max_ are shown in A panels, while adult CT_max_ is shown in B panels.

Figure S9: Change in probability of occurrence from current day to RCP 8.5 end of century. Warm colors indicate a larger reduction in thermal suitability while cool colors represent an increase in thermal suitability. Outputs are included for all seven finfish species and both average and adult CT_max_. Current marine protected areas are included as white overlayed polygons.

Figure S10: Change in suitability relative to conservation status. Trends are similar across models with MPAs suitability change largely matching the background distribution. Histograms show the MPAs have similar change in suitability estimates as the surrounding areas indicating that MPAs were not selected for their for their climate resilience. Green bars represent values within an MPA, while blue bars represent all other areas within the Caribbean.

Figure S11: Change in probability of occurrence from current day to RCP 8.5 end of century. Warm colors indicate a larger reduction in thermal suitability while cool colors represent an increase in thermal suitability. Panel A represents adult CT_max_ models while panel B represents average CT_max_ models Change maps for each species were extracted from with the MPA boundary, averaged across species and each pixel to give a singular average change value for each MPA.

Supplemental Code 1: JAGS code used to model trends in biomass as a function of change in temperature suitability from SDMs.

model {

##********************************************************
\## Level 1 Priors for subregion-level effects and variation
##********************************************************

for (k in 1:nspp){
\# Draw Subregion effects
for (subreg in 1:nsubreg){
beta.0[subreg, k] ~ dnorm(0, tau.beta.0[k])
beta.1[subreg, k] ~ dnorm(0, tau.beta.1[k])
}
}

for (k in 1:nspp){
\# Variation in abundance intercept from subregions
sigma.beta.0[k] ~ dunif(0, 100)
tau.beta.0[k] <- 1/(sigma.beta.0[k]*sigma.beta.0[k])

\# Variation in abundance slope from subregion
sigma.beta.1[k] ~ dunif(0, 100)
tau.beta.1[k] <- 1/(sigma.beta.1[k]*sigma.beta.1[k])
}

\## *******************************************************
\## Level 2 Priors for site-level coefficients and variation
\## *******************************************************

#Submodel coefficients
for (k in 1:nspp){
for (l in 1:ncols) {
coef.0[l, k] ~ dnorm(0, 0.001)
coef.1[l, k] ~ dnorm(0, 0.001)
}
}

for (k in 1:nspp){
\# Variation in abundance intercept from siteXspp
sigma.gam.0[k] ~ dunif(0, 100)
tau.gam.0[k] <- 1/(sigma.gam.0[k]*sigma.gam.0[k])

\# Variation in abundance slope from siteXspp
sigma.gam.1[k] ~ dunif(0, 100)
tau.gam.1[k] <- 1/(sigma.gam.1[k]*sigma.gam.1[k])
}

\## *******************************************************
\## Likelihood function
\## *******************************************************

for (k in 1:nspp){

\# Diffuse prior for shape parameter
r[k] ~ dgamma(0.01, 0.01)

#Level 1
for(i in 1:N) {

\# Generate total intercepts and slopes for effect of year on biomass

```
a.0[i, k] <- beta.0[subregion[i], k] + gam.0[site[i], k]
b.1[i, k] <- beta.1[subregion[i], k] + gam.1[site[i], k]

# Gamma likelihood function
    Y_1[i, k] ~ dgamma(r[k], lambda[i, k])
    lambda[i, k] <- r [k]/ mu.y[i, k]
    log(mu.y[i, k]) <- eta[i, k]
    eta[i, k] <- a.0[i, k] + b.1[i, k]*z.year[i]

} #/i

#Level 2
for(j in 1:J) {

```

#model mean biomass (intercepts) and trends (slopes) as a function of

#change in climate suitability and other covariates

gam.0[j, k] ~ dnorm(mu.gam.0[j, k], tau.gam.0[k])
mu.gam.0[j, k] <- inprod(coef.0[ , k], X[j, ,k])

gam.1[j, k] ~ dnorm(mu.gam.1[j, k], tau.gam.1[k])
mu.gam.1[j, k] <- inprod(coef.1[ , k], X[j, ,k])
}
} #/i

}
